# Identification of a Host-Targeted Compound to Control Typhoid Fever

**DOI:** 10.1128/spectrum.00619-22

**Published:** 2022-05-17

**Authors:** Ky V. Hoang, Katherine Woolard, Ching Yang, Christian Melander, John S. Gunn

**Affiliations:** a Center for Microbial Pathogenesis, Abigail Wexner Research Institute at Nationwide Children’s Hospital, Columbus, Ohio, USA; b Infectious Diseases Institute, The Ohio State University, Columbus, Ohio, USA; c Department of Chemistry and Biochemistry, University of Notre Dame, Notre Dame, Indiana, USA; d Department of Veterinary Biosciences, College of Veterinary Medicine, The Ohio State University, Columbus, Ohio, USA; e Department of Pediatrics, The Ohio State University College of Medicine, Columbus, Ohio, USA; Health Canada

**Keywords:** KH-1-2, host-targeted therapy, typhoid fever, immunomodulation, *Salmonella* Typhi

## Abstract

Typhoid fever is caused primarily by the enteric microbe Salmonella enterica serovar Typhi and remains a major global health problem with approximately 14 million new infections and 136,000 fatalities annually. While there are antibiotic options available to treat the disease, the global increase in multidrug-resistant strains necessitates alternative therapeutic options. Host-targeted therapeutics present a promising anti-infective strategy against intracellular bacterial pathogens. A cell-based assay identified a compound that inhibits *Salmonella* proliferation in infected cells, 2-(3-hydroxypropyl)-1-(3-phenoxyphenyl)-1,2-dihydrochromeno[2,3-c]pyrrole-3,9-dione (KH-1), which is devoid of direct activity against *Salmonella*. The compound inhibits the growth of both antibiotic-sensitive and -resistant *Salmonella* strains inside macrophages and reduces lactate dehydrogenase (LDH) release from *Salmonella*-infected cells. Subsequent screening of KH-1 commercial analogs identified 2-(4-fluorobenzyl)-1-(3-phenoxyphenyl)-1,2-dihydrochromeno[2,3-c] pyrrole-3,9-dione (KH-1-2), which is more effective in controlling *Salmonella* growth inside macrophages. *In vitro* KH-1-2 treatment of *Salmonella* infection resulted in an 8- to 10-fold reduction in bacterial load in infected macrophages. In combination with suboptimal ciprofloxacin treatment, KH-1-2 further reduces *Salmonella* growth inside macrophages. The toxicity and efficacy of KH-1-2 in controlling *Salmonella* infection were examined *in vivo* using a mouse model of typhoid fever. No significant compound-related clinical signs and histological findings of the liver, spleen, or kidney were observed from uninfected mice that were intraperitoneally treated with KH-1-2. KH-1-2 significantly protected mice from a lethal dose of infection by an antibiotic-resistant *Salmonella* strain. Thus, our study provides support that this is a promising lead compound for the development of a novel host-targeted therapeutic agent to control typhoid fever.

**IMPORTANCE** *Salmonella* spp. cause significant morbidity and mortality worldwide. Typhoidal spp. (e.g., *S.* Typhi) cause a systemic disease typically treated with antibiotics. However, growing antibiotic resistance is resulting in increased treatment failures. We screened a compound library for those that would reduce *Salmonella*-induced macrophage toxicity, identifying compound KH-1. KH-1 has no direct effects on the bacteria but limits *Salmonella* survival in macrophages and protects against lethal infection in a mouse model of typhoid fever. A suboptimal concentration of ciprofloxacin worked in conjunction with the compound to further decrease *Salmonella* survival in macrophages. An analog (KH-1-2) was identified that possessed increased activity *in vitro* in macrophages and *in vivo* against both antibiotic-sensitive and -resistant strains. Thus, we report the identification of a lead compound that may be a useful scaffold as a host-directed antimicrobial against typhoid fever.

## INTRODUCTION

*Salmonella* species (spp.) are Gram-negative facultative intracellular bacterial pathogens responsible for approximately 1.3 billion human infections annually worldwide (1). *Salmonella* infection results in two primary clinical manifestations: gastroenteritis and typhoid fever. Gastroenteritis is caused by nontyphoidal *Salmonella* serovars, which are the most common cause of death from diarrheal disease and are the leading cause of foodborne disease outbreaks in the United States (2). Typhoid fever is caused by Salmonella enterica serovar Typhi and various *Salmonella* Paratyphi pathovars. It is a systemic disease that leads to altered mental states, ileus, gastrointestinal bleeding, intestinal perforation, septic shock, and death. There are more than 14 million cases of typhoid fever and 136,000 deaths per year worldwide (3). *S.* Typhi is a human-restricted serovar and is unable to colonize in mice; however, the related serovar *S.* Typhimurium causes a typhoid fever-like disease in mice and is used as a model to study human typhoid fever as it recapitulates many characteristics of the human disease.

Upon infection through ingestion of contaminated food and/or water, *Salmonella* spp. reach the lower gastrointestinal tract, traverse the intestinal epithelium through the M cells, and enter the lamina propria and lymphoid follicles, where bacteria are taken up by macrophages and reside in *Salmonella*-containing vacuoles (SCVs). In typhoidal infection, the *Salmonella*-containing macrophages disseminate bacteria to common distal sites of the body, including the spleen, liver, and bone marrow (4). Virulence factors critical for the induction of proinflammatory responses in infected macrophages include pathogen-associated motifs (e.g., lipopolysaccharide [LPS], flagellin) that stimulate innate immunity and proinflammatory effectors (1). Inside SCVs, the bacteria utilize a type III secretion system to deliver many protein effectors into the cytoplasm to modulate the host immune system and alter vesicle trafficking, benefiting bacterial replication and dissemination (1, 5–7). *Salmonella* infection robustly induces proinflammatory cytokines, including tumor necrosis factor (TNF-α) and interleukin 6 (IL-6), and triggers caspase-1-dependent proinflammatory programmed cell death (8–10). The induction of the inflammatory response and bacterial-associated cell death is a bacterial strategy to promote disease (11).

The emergence of multidrug resistance is increasingly recognized among *S.* Typhi lineages and is a leading cause of treatment failure (12, 13). The current treatments for typhoid fever typically rely on antibiotics that directly target *Salmonella* (e.g., protein biosynthesis inhibition, membrane disruption, or DNA/RNA synthesis inhibition) (12) and therefore have the highest and most direct efficacy when the bacterium is growing in the extracellular environment. Once the bacterium resides intracellularly, however, it adds a level of complexity to treatment options. The *S.* Typhi multidrug-resistant strains overcome the first-line drugs for therapy, including ampicillin, chloramphenicol, and trimethoprim-sulfamethoxazole (13, 14). Epidemiological studies in Pakistan and India showed that 73.7% (*n* = 80), 56.2%, and 52.5% of *S.* Typhi isolates had developed resistance to sulfonamide, ampicillin, or streptomycin, respectively, with 58.7% of the overall isolates having multidrug resistance (15). Vaccine approaches provide variable protection from *Salmonella* infections and can cause adverse side effects (16, 17). The development of canonical antimicrobials that directly target *S.* Typhi must continue, but additional approaches are also urgently needed, including host-targeted therapy.

Host-targeted therapy can interfere with host immune pathways that are required by a pathogen for productive replication and persistence. It may also enhance the immune response by stimulating host pathways that are involved in host defense against the pathogen or those that are perturbed and disbalanced by a pathogen at the site of infection. These approaches can be used alone or in combination with traditional antibiotics. Not only does the host-directed therapeutic lessen the pathogen’s ability to evade clearance by the immune system, but it also limits the pathogen’s development of resistance since the therapeutic is not directed at the pathogen itself. In fact, host-targeted therapy has been developed to treat infections by several intracellular bacterial pathogens (18–21), viral diseases (22), and malaria (23).

The work described here details the development of a simple, cell-based, high-throughput assay to screen potential host-targeted compounds to control typhoid fever. *Salmonella* infection induces macrophage death indicated by the release of lactate dehydrogenase (LDH) in the medium. Using the LDH assay to measure cellular metabolic activity and cell viability, a subset of the ChemBridge eukaryotic kinase inhibitor/ATP-mimetic library was screened for compounds that reduce *Salmonella*-associated cell death without direct antimicrobial activity against *Salmonella in vitro*. This study provides a viable lead candidate for the continuation of host-targeted drug discovery efforts with the goal of treatment of typhoid fever caused by both antibiotic-sensitive and -resistant bacteria.

## RESULTS

### The cell-based assay screen for compounds that protect *S.* Typhimurium-infected macrophages from death.

**FIG 1:**
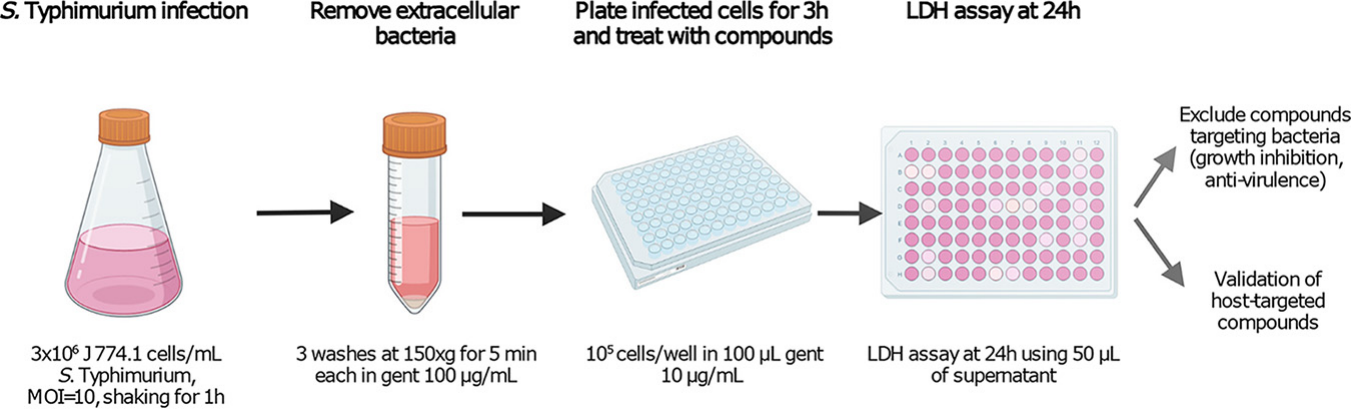
Schematic of the screen to identify the host-targeted compounds.

**FIG 2:**
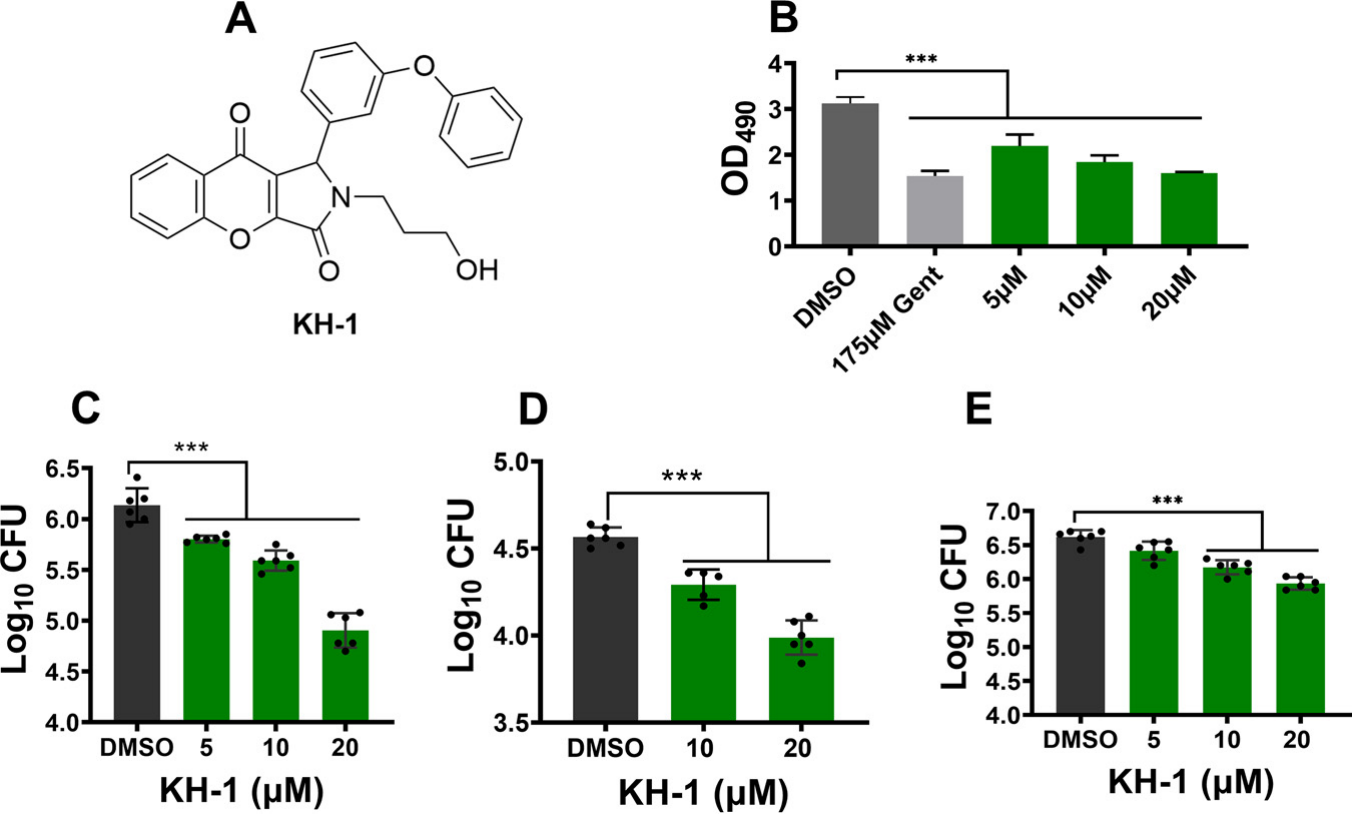
Identification of KH-1. (A) Chemical structure of KH-1. (B) KH-1 reduced LDH release from *S.* Typhimurium-infected macrophages. J774.1 macrophages were infected with *S.* Typhimurium, and the infected cells were treated with various concentrations of KH-1. The control group was treated with DMSO (negative control) or gentamicin (Gent; positive control). LDH release was evaluated at 24 h posttreatment. KH-1 inhibited the growth of wild-type *S*. Typhimurium inside the infected macrophages. J774.1 (C) or THP-1 (E) macrophages were infected with wild-type *S.* Typhimurium, and the infected cells were treated with various concentrations of KH-1. KH-1 inhibited the growth of ciprofloxacin-resistant *S.* Typhimurium. (D) J774.1 macrophages were infected with ciprofloxacin-resistant *S.* Typhimurium, and then the infected cells were treated with various concentrations of KH-1. The intracellular bacterial growth in panels C, D, and E was determined at 24 h posttreatment. The data were presented as a representative of three independent experiments. NS, not significant; *n* = 3; ***, *P* < 0.001.

We developed a cell-based assay to screen 3,000 compounds of an ATP-mimetic library purchased from ChemBridge using J774.1 macrophages for those that protect *S*. Typhimurium-infected macrophages from death. The viability of *Salmonella*-infected J774.1 cells was determined by measuring LDH release from the infected cells. The monolayer of infected macrophages was prepared in 96-well plates. Test compounds were added at the final concentration of 25 μM and remained for the duration of the experiment. At 24 h posttreatment, 50 μL of supernatant from each well was collected and screened for compounds that reduced LDH release in comparison to that of the control untreated *S*. Typhimurium-infected cells (Fig. 1). The initial screening identified eight hits, and subsequent rescreening using a 24-well plate format narrowed the list to three compounds. The most promising compound was 2-(3-hydroxypropyl)-1-(3-phenoxyphenyl)-1,2-dihydrochromeno[2,3-c] pyrrole-3,9-dione, which we named KH-1 (Fig. 2A) and which reduced LDH release from *S.* Typhimurium-infected macrophages in a dose-dependent manner (Fig. 2B). To examine whether the reduction in LDH release was due to the limited growth of the bacteria intracellularly, the infected cells were treated with various concentrations of KH-1, and the intracellular bacterial load was determined at 24 h posttreatment. As shown in Fig. 2C and D, KH-1 reduced intracellular bacterial growth of both antibiotic-sensitive and ciprofloxacin-resistant strains, respectively, in a dose-dependent manner. The intracellular antibacterial effects of KH-1 are not dependent on host cell species since the compound also limits *S*. Typhimurium growth in THP-1 human macrophages (Fig. 2E).

### KH-1 is not antibacterial in standard medium and does not affect *Salmonella* entry into macrophages.

**FIG 3:**
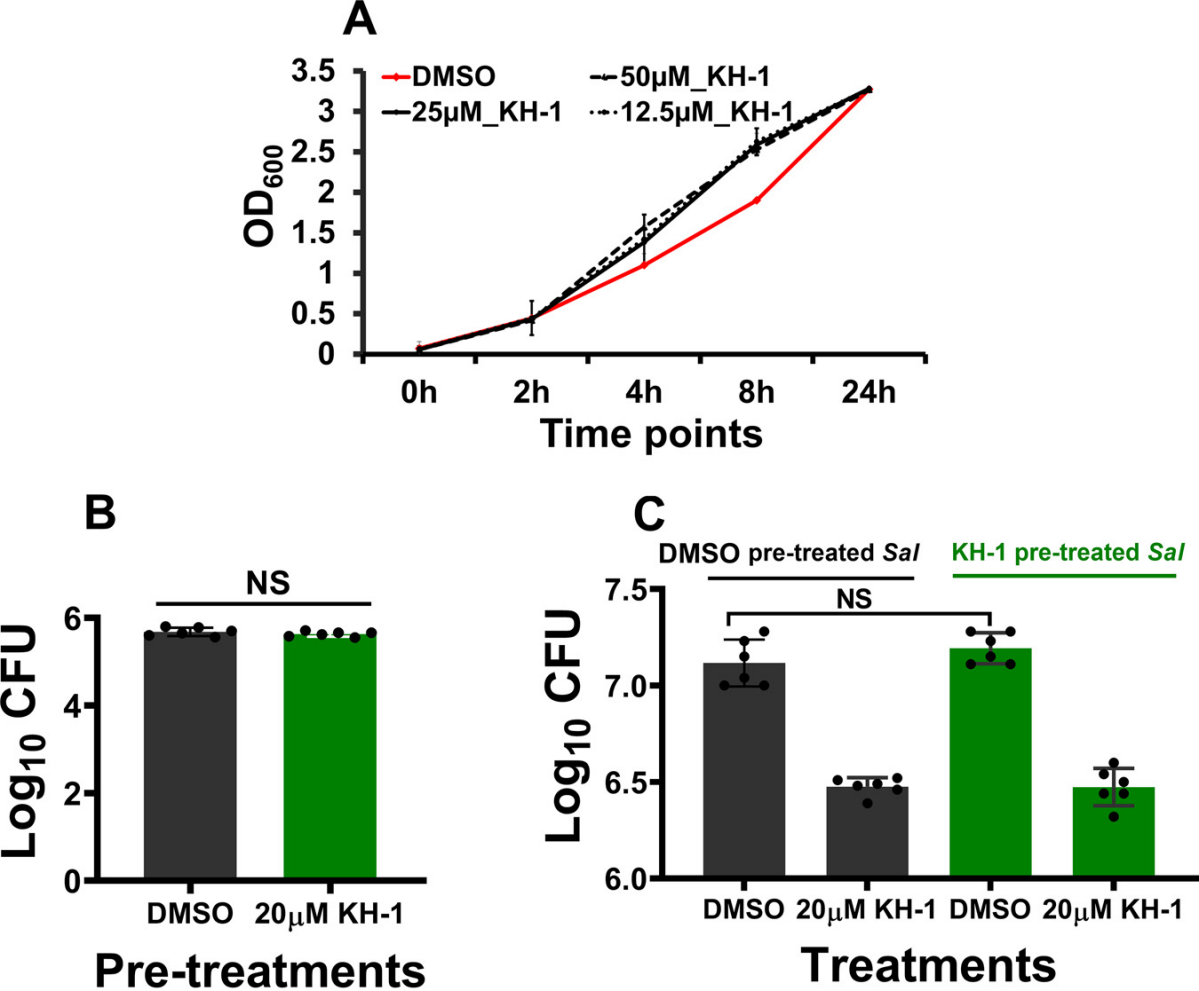
(A) KH-1 does not target *Salmonella* directly. *S.* Typhimurium was grown in Luria-Bertani (LB) broth supplemented with various concentrations of KH-1. The bacterial growth was examined at the indicated time points by measuring optical density at 600 nm (OD_600_) (*n* = 3). KH-1 pretreatment does not affect *Salmonella* virulence. *S*. Typhimurium grown in LB with 20 μM KH-1 or DMSO vehicle was used to infect J774.1 macrophages. The intracellular bacteria were recovered at 0 (B) and 24 h (C) postinfection. *n* = 3; NS, not significant.

To test whether KH-1 has direct inhibitory effects on bacteria in broth, we examined bacterial growth in the presence of various concentrations of KH-1. As shown in Fig. 3A, even with a concentration of 50 μM, which is 10-fold above the lowest concentration showing an effect on LDH release, KH-1 does not exert direct inhibitory effects on the growth of *S.* Typhimurium *in vitro*. It is also possible that the compound may negatively affect the entry of *Salmonella* into macrophages. To test this hypothesis, we precultured *S.* Typhimurium bacteria in LB containing 20 μM KH-1 and then compared their infectivity with that of bacteria that were precultured in LB without KH-1. As shown in Fig. 3B, there is no significant difference in infectivity between bacteria precultured in KH-1 and bacteria precultured in LB alone. In addition, there is no difference in the intracellular growth of precultured bacteria in KH-1 and LB alone at 24 h postinfection (Fig. 3C). These data suggested that preexposing *Salmonella* to KH-1 does not alter the invasive ability and intracellular growth of the bacteria. Since KH-1 does not directly inhibit the growth or ability of the bacteria to invade macrophages but does reduce bacterial growth intracellularly, we hypothesized that the compound modulates the host cell to limit intracellular bacterial survival.

### KH-1 sensitizes bacteria to ciprofloxacin in the infected macrophages but not in a standard medium.

**FIG 4:**
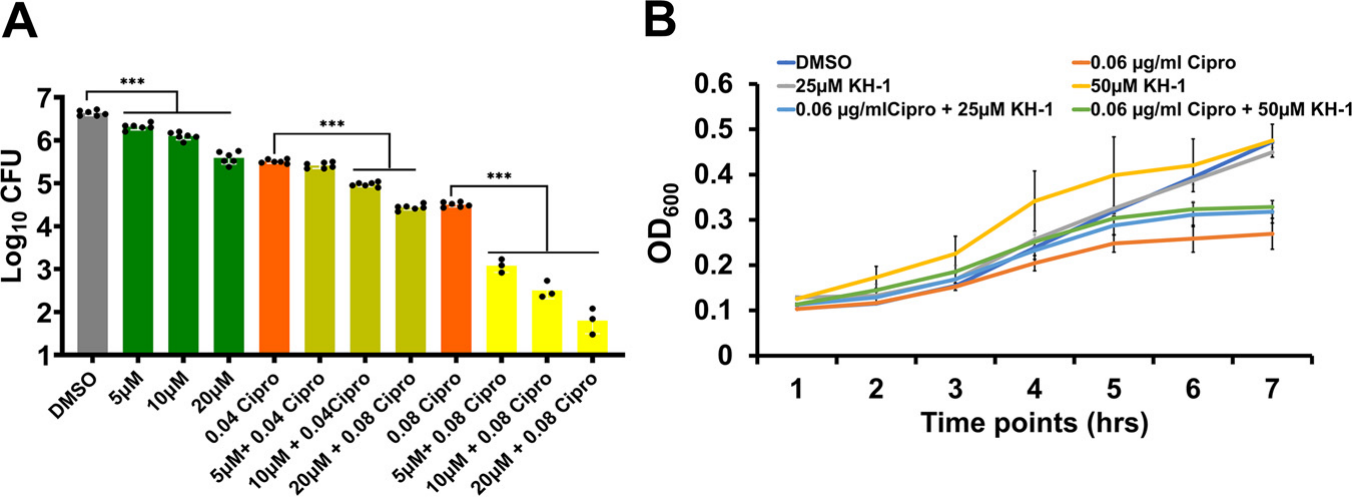
KH-1 sensitizes bacteria to ciprofloxacin in the infected macrophages but not in a standard medium. (A) *S*. Typhimurium-infected J774.1 macrophages were treated with various concentrations of KH-1 with or without suboptimal doses of ciprofloxacin. Intracellular bacteria were recovered at 24 h posttreatment. (B) *S*. Typhimurium was grown in LB broth with various concentrations of KH-1 with or without a suboptimal dose of ciprofloxacin (0.08 μg/mL). The bacterial growth was evaluated by measuring OD_600_ at different time points. *n* = 3; ***, *P < *0.001.

We next addressed whether the lead compound could be used in combination with ciprofloxacin, a common antibiotic used to treat typhoid fever. To this end, we treated *Salmonella*-infected cells with various concentrations of KH-1 and a suboptimal dose of ciprofloxacin (determined before the experiments) and determined the intracellular bacterial load at 24 h posttreatment. As shown in Fig. 4A, KH-1 treatment with suboptimal doses of ciprofloxacin consistently reduced intracellular bacterial growth to a greater extent than KH-1 alone. Importantly, KH-1 treatment did not sensitize the bacteria to ciprofloxacin in a standard bacterial culture medium (Fig. 4B). These data further demonstrate the potential of KH-1 in treating *Salmonella* systemic infection.

### Screening for more potent KH-1 analogs identified KH-1-2.

**FIG 5:**
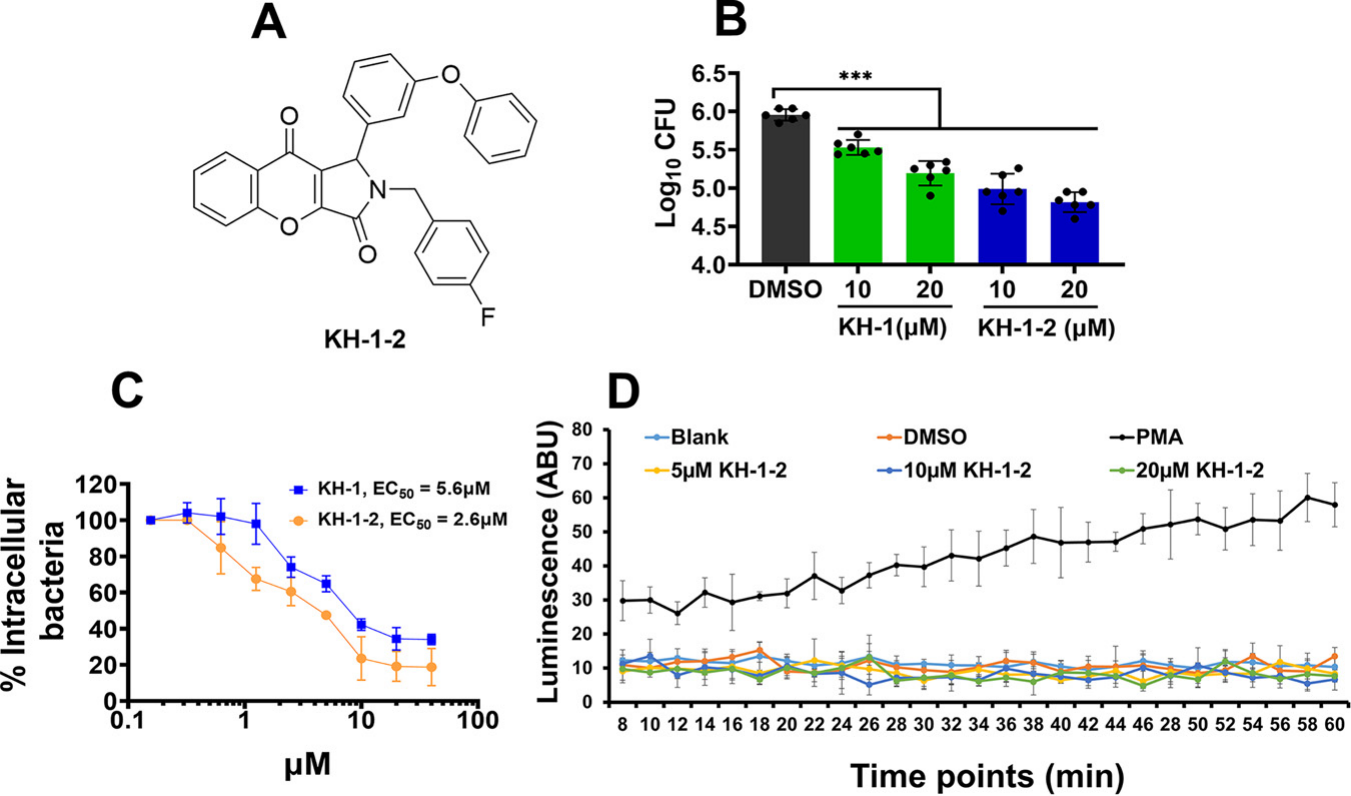
Examination of KH-1-2. (A) Chemical structure of KH-1-2. KH-1-2 has enhanced anti-*Salmonella* activity. (B) *S. Typhi*-infected J774.1 macrophages were treated with various concentrations of KH-1 or KH-1-2. Intracellular bacteria were recovered at 24 h posttreatment. (C) *S*. Typhimurium-infected J774.1 cells were treated with different concentrations of KH-1, KH-1-2, or control DMSO. Intracellular bacteria were recovered at 24 h posttreatment. The reduction in bacterial growth was calculated as percentage of CFU recovered from treated group to the DMSO control. (D) KH-1-2 does not induce ROS production in neutrophils. PLB-985 neutrophil-like cells were treated with different concentrations of KH-1-2. The ROS production was monitored at different time points. The positive-control group was treated with 0.1 μg PMA/mL. The data presented in panels B, C, and D are representative of at least three independent experiments. *n* = 3; ***, *P* < 0.001.

To improve the activity of KH-1, we performed an online search for potential analogs and experimentally examined identified compounds for toxicity and *Salmonella* intramacrophage survival. From screening four potential analogs (see Fig. S2A in the supplemental material), we identified a compound {2-(4-fluorobenzyl)-1-(3-phenoxyphenyl)-1,2-dihydrochromeno[2,3-c] pyrrole-3,9-dione} that we named KH-1-2 (Fig. 5A) that is more effective at reducing the intracellular growth of *S.* Typhi (Fig. 5B) and *S.* Typhimurium (Fig. 5C) than KH-1. As shown in Fig. 5C, KH-1-2 has a half maximal effective concentration (EC_50_) of 2.6 μM in comparison with the EC_50_ of 5.6 μM KH-1 on *S.* Typhimurium. The other three compounds tested were less effective than KH-1 (Fig. S2B).

Induction of reactive oxygen species (ROS) production is a potent mechanism for targeting infection, but prolonged excessive intracellular ROS production can lead to activation of cell death (24). To determine if KH-1-2 addition activated ROS, PLB-985 neutrophil-like cells were treated with various concentrations of KH-1-2. These data demonstrate that the host-mediated anti-*Salmonella* activity of KH-1-2 is not involved in induction of ROS production (Fig. 5D). KH-1-2 alone is also not toxic to macrophages at the effective concentrations as measured by LDH release (Fig. S2C).

### Evaluation of toxicity in mice following KH-1-2 administration.

**FIG 6:**
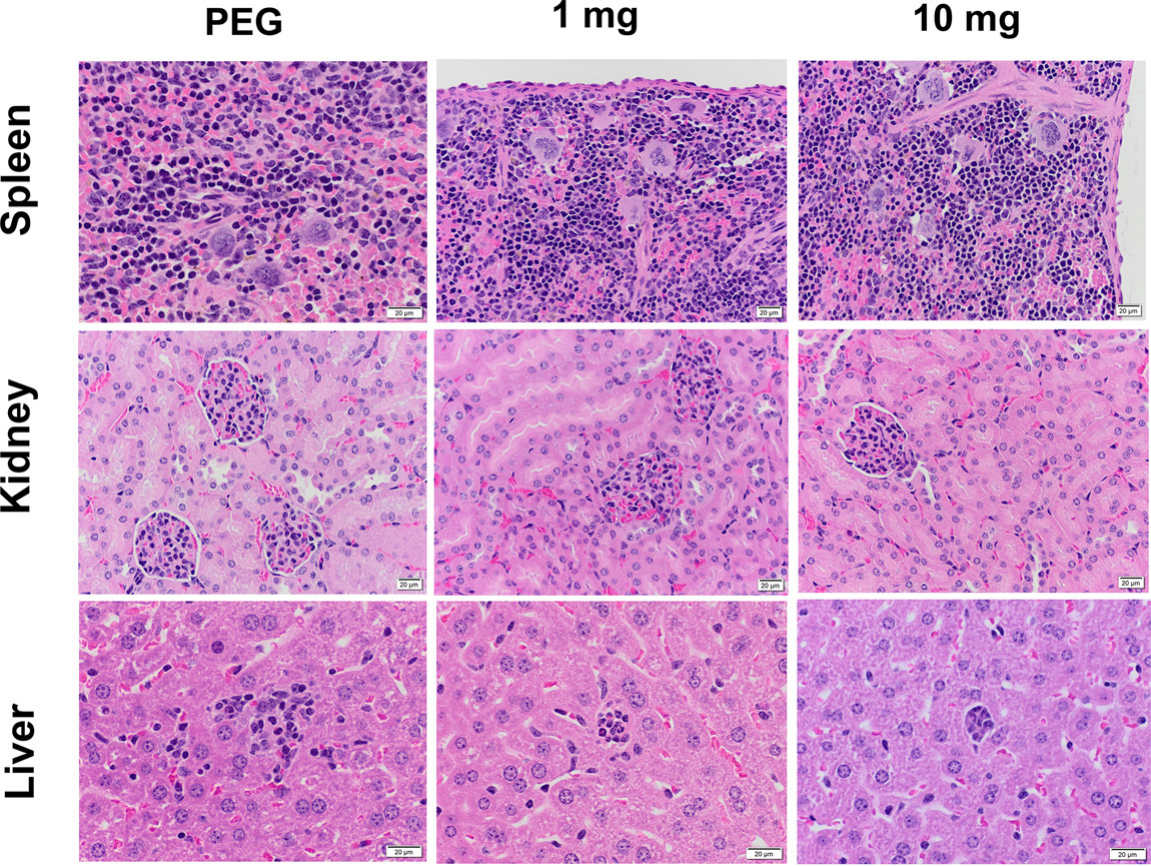
Histopathological studies of organs from mice treated with KH-1-2 for 10 consecutive days via i.p. route (×20 magnification). At the end of the experiment, mice were sacrificed, and liver, spleen, and kidney were collected, fixed in 4% paraformaldehyde for 72 h, processed, and stained with hematoxylin and eosin (H&E). No increase in immune cell migration and no significant apoptosis was observed in the spleen and kidney between the control and KH-1-2 treatment groups. For the liver histopathology studies, KH-1-2 treatment at 1 mg resulted in no adverse effects, while treatment at 10 mg resulted in minimal effects indicated by infiltration of lymphocytic cells and neutrophils.

To examine the *in vivo* toxicity of KH-1-2, uninfected mice were given KH-1-2 intraperitoneally in 200 μL of polyethylene glycol 400 (PEG 400)-0.9% saline-ethanol (50:35:15) at 1 and 10 mg/kg body weight daily for 12 consecutive days. Mice in the control group were treated with equal amounts of diluent. Mice were monitored for clinical signs, and at day 13, the liver, spleen, and kidney were collected for histopathologic evaluation. KH-1-2 treatment resulted in no adverse clinical signs (data not shown) and no dose-related macroscopic or microscopic findings upon histologic evaluation of the liver, spleen, and kidney (Fig. 6). No increase in immune cell migration or significant apoptosis in these organs was observed in treated mice. For the histopathology study of the liver, KH-1-2 treatment at 1 mg/kg resulted in no adverse effects; however, treatment at 10 mg/kg resulted in minimal alterations, including increased infiltration of lymphocytic cells and neutrophils. Overall, these data indicate that the intraperitoneal (i.p.) route of delivery of 1 and 10 mg/kg of KH-1-2 is well-tolerated in mice.

### KH-1-2 treatment of mice infected with an antibiotic-resistant *S.* Typhimurium isolate protects against lethality.

**FIG 7:**
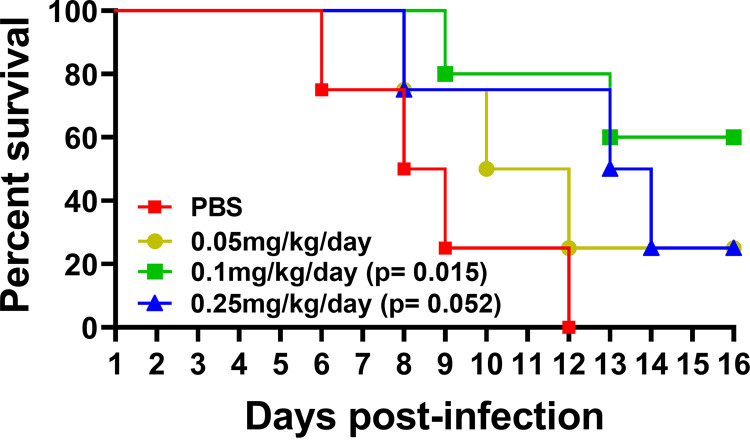
KH-1-2 treatment protects mice from lethal *S.* Typhimurium infection. Mice (4 or 5 mice per group) were orally infected with a lethal dose (10^6^ CFU/mouse) of ciprofloxacin-resistant *S.* Typhimurium. One day postinfection, the infected mice were given KH-1-2 prepared in 200 μL PBS at 0.05, 0.1, and 0.25 mg/kg per day via the intraperitoneal route for 14 consecutive days. The infected mice were monitored for survival for up to 2 weeks postinfection. DPI, days postinfection; *, *P < *0.05 with respect to PBS control.

We next sought to examine the effectiveness of delivering KH-1-2 for controlling a ciprofloxacin-resistant *S*. Typhimurium isolate in a mouse model of typhoid fever. Mice were challenged with a lethal dose of bacteria via oral gavage, and the infected mice were i.p. treated with KH-1-2 prepared in 200 μL phosphate-buffered saline (PBS) at 0.05, 0.1, and 0.25 mg/kg body weight per day for 14 consecutive days. These concentrations were driven by experiments in mice with the parent compound KH-1 (Fig. S3), which showed protection or increased time to death at an ~10-fold higher range of the compound. As expected, all mice in the control group were moribund before or at day 12 postinfection. We achieved significantly greater survival with KH-1-2 treatment groups than with the control untreated group (*P* < 0.05), with all doses providing some protection from death (Fig. 7). The dose of 0.1 mg/kg body weight/day was most effective, showing 60% survival.

## DISCUSSION

In this study, we attempted to identify new host-targeted therapeutic candidates for the treatment of typhoid fever caused by both antibiotic-sensitive and -resistant bacterial strains. The treatment of typhoid fever is confined to a few antibiotic options, including ciprofloxacin, azithromycin, and ceftriaxone. However, recent epidemiological studies reported the development of resistance to these drugs, making treatment failure too frequent (13, 25–27). There are several typhoid fever vaccines available for adults and children, but they provide variable protection and can cause side effects (28). New antimicrobial discovery has been lacking, and the incidence of *Salmonella* infections and the rapid emergence of multidrug-resistant *S.* Typhi (29) have increased, highlighting the worldwide threat of this pathogen and the urgent need for new approaches for therapeutic intervention (13).

Modulation of the host immune system as well as targeting host factors employed by pathogenic microbes for infection and survival are emerging approaches to control intracellular pathogens (30, 31). *Salmonella* spp. have evolved multiple mechanisms to counteract and exploit host immune pathways to evade killing, subsequently inducing cell death (10, 32–36) and the induction of the inflammatory response to promote disease (11). Thus, drugs that prevent *Salmonella*-induced cell death could be used as a therapy to control typhoid fever.

Host cell death assay has been developed for the screening of or evaluation of active molecules against microbial pathogens (37). Here, we report the development of a simple, cell-based assay using lactate hydrogenase (LDH) as a readout to identify compounds that protect *Salmonella*-infected macrophages from death. An initial screening of a 3,000-compound ATP-mimetic library, a subset of the ChemBridge ATP-mimetic (kinase inhibitor) library, identified eight active compounds that inhibited *Salmonella*-mediated cell death. Subsequent screening of the eight compounds focused on KH-1 (Fig. 2A), which reduced LDH release and inhibited *Salmonella* growth inside infected cells (Fig. 2C to E) with an EC_50_ in the single-digit micromolar range (Fig. 5B).

Preexposure of *Salmonella* to KH-1 does not alter bacterial invasion (Fig. 3B) or proliferation inside macrophages (Fig. 3C). That, combined with the observation that the compound does not directly inhibit bacterial growth in broth (Fig. 3A), suggests the lack of direct bacterial target for KH-1. The compound does not affect the host cell proliferation (Fig. S4) or inhibit bacterial invasion (Fig. S5). The compound also acts in an additive and/or synergistic mode with a suboptimal dose of ciprofloxacin in the infected macrophage (Fig. 4A) but not in the broth (Fig. 4B). Together, these data indicate that a host-directed mechanism of the compound is responsible for its anti-*Salmonella* activity.

Our study showed that the compound is effective in both mouse (Fig. 2C and D) and human macrophages (Fig. 2E), suggesting that it targets a common pathway in humans and mice. Additionally, the host-targeted activity of the compound was confirmed against an antibiotic-resistant clinical isolate (Fig. 2D). With the increase in multidrug-resistant (MDR) infections coupled with the lack of investment in novel antibacterials by pharmaceutical companies, combinatorial treatment regimens using multiple complementary approaches, including host-targeted therapy in combination with conventional antibiotics, are likely going to be indispensable to controlling and eradicating *Salmonella* infections. Our study showed that when combined with suboptimal doses of antibiotics, KH-1 further inhibited intracellular bacterial growth inside the macrophages (Fig. 4A), suggesting the continued study of a dual treatment regimen of the compound with antibiotics to control typhoid fever.

We examined the toxicity and therapeutic efficacy of KH-1-2 by using a mouse model. No adverse clinical signs were observed from KH-1-2-treated mice at a concentration up to 10 mg/kg/day for 10 days. Histopathological studies of liver, spleen, and kidney from KH-1-2-treated mice showed a small increase in infiltration of immune cells in the liver but not in the other two organs compared to that in control mice (Fig. 6). Thus, KH-1-2 does not appear to have significant toxicities. A more comprehensive evaluation of the potential adverse effects of KH-1-2 *in vivo* is planned for future experiments. We then used KH-1-2 to treat typhoid fever in a mouse model using a ciprofloxacin-resistant *Salmonella* strain. KH-1-2 was delivered from days 1 to 12 postinfection via i.p. route. Mice in KH-1-2-treated groups (0.05 mg/kg to 0.25 mg/kg) were protected from lethal infection (Fig. 7). These effective doses were much lower than the doses showing no or minimal toxicity dose (1 to 10 mg/kg), suggesting a potential wide therapeutic window of the compound. However, we observed that a higher dose of the compound provided less protective efficacy, perhaps due to partial agonism/antagonism activity of the target or off-target effects. More comprehensive optimization of the effective dose and delivery route is under investigation.

Reactive oxygen species (ROS) produced by the host plays an important role in controlling microbial infection, including in those infected by *Salmonella* (38, 39). Paradoxically, dysregulation or excessive production of ROS induces damage to the host and promotes particular infections (40). Our data showed that KH-1-2 does not induce ROS production (Fig. 5D). The cellular mechanisms that are targeted by KH-1-2, result in the reduction of *Salmonella* growth inside the macrophages, and increase survival of infected mice are unknown but are actively being elucidated.

In summary, the work presented describes the application of a cell-based throughput assay system using an LDH assay approach to screen a chemical library for host-targeted compounds active against *Salmonella*. These studies provide the support that KH-1-2 may be a promising lead compound for the development of new host-targeted therapeutic agents to control typhoid fever caused by both antibiotic-sensitive and -resistant *Salmonella* strains.

## MATERIALS AND METHODS

### Cells and bacterial strains.

J774.1 murine macrophages were cultured in Dulbecco modified Eagle medium (DMEM; Gibco-Life Technologies, Grand Island, NY). THP-1 human macrophages and PLB-985 cells (41) were cultured in RPMI 1640 (Gibco-Life Technologies, Grand Island, NY). Cells were maintained under humidified conditions at 37°C, 5% CO_2_ in medium supplemented with 10% fetal bovine serum (FBS; GIBCO-BRL) and penicillin-streptomycin (Gibco-Life Technologies, Grand Island, NY; 100 μg/mL each). The *S.* Typhimurium wild-type strain ATCC 14028 and an *S.* Typhi strain, TY2, were used in this study. A clinical ciprofloxacin-resistant *S.* Typhimurium isolate was collected from Ethiopia (42). All bacterial strains were cultured in Luria-Bertani (LB) broth (Difco, Detroit, MI) and incubated at 37°C with aeration.

### Compound library, reference compounds, and reagents.

A 3,000-member ATP-mimetic library in 96-well plate format was sourced from ChemBridge. The reference compounds 2-(3-hydroxypropyl)-1-(3-phenoxyphenyl)-1,2-dihydrochromeno[2,3-c] pyrrole-3,9-dione (KH-1) and 2-(4-fluorobenzyl)-1-(3-phenoxyphenyl)-1,2-dihydrochromeno[2,3-c] pyrrole-3,9-dione (KH-1-2) were purchased from ChemBridge. KH-1-2 was resynthesized in-house to confirm structure, purity, and activity. All commercial solvents and reagents were purchased from VWR or Sigma-Aldrich and used without any further purification. Reactions were monitored using thin-layer chromatography (TLC) using glass-backed precoated silica gel plates from VWR (TLC silica gel 60 sheets, Millipore Sigma, F254, 60 Å pore 230 to 400 mesh) using UV visualization. Column chromatography was performed using silica gel (60 Å, particle size 40 to 60 μm, VWR). Deuterated solvents for nuclear magnetic resonance (NMR) characterization were purchased from Cambridge Analytical via VWR and used as-is. All NMR spectra were performed at room temperature and recorded on a Bruker AVANCE III HD 400 Nanobay spectrometer without the use of signal suppression function and calibrated using the residual undeuterated solvent peak (chloroform-d: δ 7.26 ppm ^1^H NMR, 77.16 ppm 13C NMR). Proton (^1^H) NMR data are reported as follows: chemical shift in ppm (multiplicity, coupling constant[s] in Hz, relative integration). Abbreviations used are s, singlet; d, doublet; t, triplet; q, quartet; m, multiplet. High-resolution mass spectra (HRMS) were recorded on a Bruker micrOTOF II by electrospray ionization (ESI) time of flight (TOF) experiments using direct infusion in 9:1 acetonitrile/water. Analyses were performed by the Mass Spectrometry and Proteomics Facility at the University of Notre Dame and reported as *m/z*. KH-1-2 was characterized and tested at ≥95% purity as determined by liquid chromatography on a Bruker micrOTOF-Q II by the University of Notre Dame Mass Spectrometry and Proteomics Facility.

### Synthesis of KH-1-2.

The KH-1-2 synthesis scheme is depicted in Fig. S1. Methyl 4-(2-hydroxyphenyl)-2,4-dioxobutanoate was synthesized as reported previously (43) from dimethyl oxalate and 2-hydroxyacetophenone. Following literature precedent (44), we added to an oven-dried 10 mL round bottom flask 4-fluorobenzylamine (80 mg, 0.40 mmol, 1.0 eq) and 4 Å molecular sieves in 1.25 mL dichloromethane. Anhydrous pyrrolidine (3.3 μL, 40.0 μmol, 0.10 eq) was added directly to the reaction followed by 3-phenoxybenzaldehyde (60 mg, 0.48 mmol, 1.2 eq). The reaction was stirred for 30 min at room temperature and monitored by TLC. The resulting solution was filtered, dried over anhydrous sodium sulfate, filtered, and evaporated *in vacuo* to yield (*E*)*-N-*(4-fluorobenzyl)-1-(3-phenoxyphenyl)methanimine. This crude oil was used directly in the next reaction without further purification.

Following the literature procedure for 1,2-diaryl-1,2-dihydrochromeno[2,3-*c*]pyrrole-3,9-diones (45), methyl 4-(2-hydroxyphenyl)02,4-dioxobutanoate (90 mg, 0.40 mmol, 1 eq) and (*E*)*-N-*(4-fluorobenzyl)-1-(3-phenoxyphenyl)methanimine (122 mg, 0.40 mmol, 1 eq) were added in 5 mL glacial acetic acid to a 10-mL round bottom flask. The reaction was heated to reflux for 30 min while stirring. The mixture was cooled, slowly neutralized with cold methanol, and evaporated *in vacuo* to yield a crude oil. This oil was redissolved in dichloromethane, washed with saturated sodium chloride, dried over anhydrous sodium sulfate, filtered, and evaporated *in vacuo*. The compound was purified using flash column chromatography with a gradient of 100% hexanes to 50% hexanes/50% ethyl acetate to yield KH-1-2 as an off-white solid (25% yield, 99% pure). ^1^H NMR (400 MHz, chloroform-d) δ 8.16 (dd, *J *= 8.0, 1.7 Hz, 1H), 7.78 to 7.68 (m, 2H), 7.46 (ddd, *J *= 8.2, 6.8, 1.4 Hz, 1H), 7.34 (dt, *J *= 9.4, 7.7 Hz, 3H), 7.18 to 7.11 (m, 3H), 7.03 to 6.98 (m, 4H), 6.97 (t, *J *= 1.4 Hz, 1H), 6.95 (d, *J *= 2.0 Hz, 1H), 6.88 (t, *J *= 2.0 Hz, 1H), 5.23 (d, *J *= 14.4 Hz, 2H), 3.83 (d, *J *= 14.9 Hz, 1H); ^13^C NMR (101 MHz, chloroform-d) δ 173.2, 162.5 (d, *J *= 247.2 Hz), 162.0, 161.3, 158.2, 158.1, 156.5, 154.4, 154.1, 134.8, 134.6, 131.7, 131.7, 130.6, 130.4, 130.3, 129.9, 127.7, 126.1, 125.5, 123.8, 122.2, 119.2, 119.1, 119.1, 118.0, 116.0, 115.8, 59.2, 43.7; IR *V*_max_ (cm^−1^) 3,066, 3,047, 2,916, 2,857, 1,711, 1,652, 752; HRMS *m/z* calculated for C_30_H_21_FNO_4_ [M+H]^+^: 478.1449, found 478.1437.

### Cell-based infection screening assay.

The strategy to identify host-targeted compounds is outlined in Fig. 1. Briefly, J774.1 macrophages in suspension at 3 × 10^6^ cells/mL were infected with *S*. Typhimurium at a multiplicity of infection (MOI) of 10 for 1 h with orbital shaking at 80 rpm. The extracellular bacteria were eliminated and removed by the addition of 100 μg/mL gentamicin to the culture medium for 30 min and then washed three times using DMEM by centrifugation at 160 × *g* for 10 min each. The infected macrophages were resuspended in a medium containing 10 μg/mL gentamicin, seeded onto 96-well plates at 10^5^ cells/well in 150 μL medium, and allowed to adhere to the well for 3 h. The infected cells in each well were then treated with 25 μM concentrations of each compound from the ATP-mimetic library using a multichannel pipette. The positive-control well was treated with 170 μg/mL gentamicin which exhibits lethality to intracellular bacteria with prolonged incubation. After 24 h of incubation, an LDH assay (Roche Applied Science, Indianapolis, IN) was performed using 50 μL of supernatant from each well. Absorbance at 570 nm was determined via a plate reader Spectra Max M3. The compounds that reduced LDH release from *Salmonella-*infected macrophages compared with that from untreated wells were selected for subsequent screening and tested for a lack of direct killing of *Salmonella*.

### Broth antibacterial assay.

Overnight *Salmonella* cultures were subcultured (1:50) in fresh LB broth containing various concentrations of the compound or in combination with a suboptimal dose of ciprofloxacin and incubated at 37°C with aeration. An equivalent amount of dimethyl sulfoxide (DMSO) was added in a control group. The bacterial growth was monitored at an optical density at 600 nm (OD_600_) at the indicated time points using plate reader Spectra Max M3.

### Neutrophil reactive oxygen species production assay.

PLB-985 cells that were cultured in RPMI supplemented with 10% FBS and penicillin-streptomycin (100 μg/mL each) were differentiated to a neutrophil-like phenotype by 6-day incubation in RPMI supplemented with 0.5% *N*,*N*-dimethylformamide, 0.5% FBS, 1% Nutridoma-SP (Roche; Mannheim, Germany), 2 mM l-glutamine, and 1× penicillin/streptomycin (46). Medium was replaced on day 3. On day 6 after differentiation, the medium was removed and replaced with 100 μL fresh medium containing different concentrations of KH-1-2. Cells in the positive-control group were stimulated with 400 ng phorbol myristate acetate (PMA)/mL. Cells in the negative-control group were treated with an equivalent concentration of DMSO. All groups were supplied with luminol (final concentration of 500 μM), and reactive oxygen species (ROS) production at 37°C was monitored in triplicate by luminol-dependent chemiluminescence measured every 2 min for 1 h using a Spectra Max M3 plate reader.

### Bacterial cultures and analysis of bacterial growth in macrophages.

Analysis of the inhibitory effects of the compounds on bacterial growth in macrophages was performed as described previously (20). Briefly, overnight cultures of *S*. Typhimurium or *S.* Typhi were subcultured (1:50) in fresh LB broth and incubated for 4 h at 37°C with aeration. Bacteria were then collected by centrifugation at 3,000 × *g* for 10 min and suspended in phosphate-buffered saline (PBS) to an optical density of 0.6 at 600 nm (5 × 10^8^ CFU/mL). J774.1 or THP-1 cells were infected with *Salmonella* strains at an MOI of 10 in the presence of 10% serum in DMEM and RPMI 1640, respectively (Gibco-Life Technologies). One hour after infection, extracellular bacteria were removed by the addition of 100 μg/mL gentamicin to the culture medium for 30 min, and the cell layer was thoroughly washed three times with prewarmed PBS at 37°C. The infected cells were then treated with different concentrations of each compound or in combination with a suboptimal dose of ciprofloxacin in a fresh culture medium containing 10% FBS and 10 μg/mL gentamicin that inhibited potential reinfection by extracellular bacteria. At 24 h posttreatment, the infected cells were lysed with 0.1% Triton X-100 (Calbiochem, San Diego, CA) in PBS for 10 min. The cell lysates were then serially diluted with PBS and drip plated on LB agar plates. The intracellular bacterial loads were determined by enumerating CFU after 24 h of incubation at 37°C.

### Mice.

Pathogen-free 7- to 8-week-old female BALB/c mice were purchased from Jackson Laboratory. Mice were provided food and water *ad libitum*, divided into groups in sterile microisolator cages, and allowed to acclimate for 2 to 3 days before the experiments. All experimental procedures were performed in strict accordance with guidelines established by Nationwide Children’s Hospital Institutional Animal Care and Use Committees (IACUCs), and all efforts were made to minimize animal discomfort.

### Toxicity study of KH-1-2 *in vivo*.

To examine the toxicity of KH-1-2, mice were intraperitoneally (i.p.) given KH-1-2 that was dissolved in 200 μL of polyethylene glycol 400 (PEG 400)-0.9% saline-ethanol (50:35:15) at 1 mg and 10 mg/kg of body weight per day for 12 consecutive days. Mice in the control groups received 200 μL of PEG-saline-ethanol. The experimental animals were observed daily throughout the study for clinical signs and mortality. At the end of the experiment, mice were sacrificed, and liver, spleen, and kidney were collected, fixed in 4% paraformaldehyde for 72 h, processed, and stained with hematoxylin and eosin (H&E) for histopathologic evaluation, which was performed at the Morphology Core at Nationwide Children’s Hospital.

### Mouse model of typhoid fever and evaluation of protective efficacy of KH-1-2.

*S.* Typhi is a human-restricted pathogen and is unable to colonize in mice. However, *S.* Typhimurium causes a typhoid fever-like disease in mice and is widely used as a model to study human typhoid fever (47). Briefly, an overnight culture of a ciprofloxacin-resistant *S*. Typhimurium strain was subcultured (1:50) in fresh LB broth and incubated for 6 h at 37°C with aeration. Bacteria were then collected by centrifugation, suspended in PBS to an optical density of 0.6 at 600 nm (5 × 10^8^ CFU/mL), and then diluted to a concentration of 5 × 10^6^ CFU/mL in PBS. Mice were infected with the ciprofloxacin-resistant *S*. Typhimurium at a lethal dose of 10^6^ CFU per mouse in 200 μL PBS via the i.p. route.

After determining the maximum tolerable dose of KH-1-2 by the i.p. route (10 mg/kg of body weight per day), the protective efficacy of KH-1-2 as a treatment for typhoid fever was evaluated. Mice (4 or 5 mice per group) were infected at day 0 with a lethal dose of the ciprofloxacin-resistant *S.* Typhimurium isolate as described above. One day postinfection, the infected mice were given KH-1-2 prepared in 200 μL PBS at 0.05, 0.1, and 0.25 mg/kg body weight per day via i.p. delivery for 14 consecutive days.

### Statistical analysis.

Data are presented as mean ± standard deviation (SD). *P* values were calculated using one-way analysis of variance (ANOVA) for multiple comparisons and adjusted with Bonferroni’s correction or using a nonpaired Student’s *t* test, where two group means were compared; *, *P* < 0.05; **, *P* < 0.01; ***, *P* < 0.001; NS, not significant. For the animal experiments, *P* values were calculated using log-rank (Mantel-Cox) test with respect to the PBS control. Statistical analysis was performed using GraphPad Prism 9.

## References

[1] Coburn B, Grassl GA, Finlay BB. 2007. *Salmonella*, the host and disease: a brief review. Immunol Cell Biol 85:112–118. doi:10.1038/sj.icb.7100007. 17146467

[2] Mead PS, Slutsker L, Dietz V, McCaig LF, Bresee JS, Shapiro C, Griffin PM, Tauxe RV. 1999. Food-related illness and death in the United States. Emerg Infect Dis 5:607–625. doi:10.3201/eid0505.990502. 10511517 PMC2627714

[3] Typhoid GBD, Paratyphoid C. 2019. The global burden of typhoid and paratyphoid fevers: a systematic analysis for the Global Burden of Disease Study 2017. Lancet Infect Dis 19:369–381. doi:10.1016/S1473-3099(18)30685-6. 30792131 PMC6437314

[4] Dougan G, John V, Palmer S, Mastroeni P. 2011. Immunity to salmonellosis. Immunol Rev 240:196–210. doi:10.1111/j.1600-065X.2010.00999.x. 21349095

[5] Jennings E, Thurston TLM, Holden DW. 2017. Salmonella SPI-2 type III secretion system effectors: molecular mechanisms and physiological consequences. Cell Host Microbe 22:217–231. doi:10.1016/j.chom.2017.07.009. 28799907

[6] Lian H, Jiang K, Tong M, Chen Z, Liu X, Galán JE, Gao X. 2021. The *Salmonella* effector protein SopD targets Rab8 to positively and negatively modulate the inflammatory response. Nat Microbiol 6:658–671. doi:10.1038/s41564-021-00866-3. 33603205 PMC8085087

[7] Hahn M, Covarrubias-Pinto A, Herhaus L, Satpathy S, Klann K, Boyle KB, Münch C, Rajalingam K, Randow F, Choudhary C, Dikic I. 2021. SIK2 orchestrates actin-dependent host response upon *Salmonella* infection. Proc Natl Acad Sci USA 118:e2024144118. doi:10.1073/pnas.2024144118. 33947818 PMC8126862

[8] Hersh D, Monack DM, Smith MR, Ghori N, Falkow S, Zychlinsky A. 1999. The Salmonella invasin SipB induces macrophage apoptosis by binding to caspase-1. Proc Natl Acad Sci USA 96:2396–2401. doi:10.1073/pnas.96.5.2396. 10051653 PMC26795

[9] Wemyss MA, Pearson JS. 2019. Host cell death responses to non-typhoidal *Salmonella* infection. Front Immunol 10:1758. doi:10.3389/fimmu.2019.01758. 31402916 PMC6676415

[10] Fink SL, Cookson BT. 2007. Pyroptosis and host cell death responses during *Salmonella* infection. Cell Microbiol 9:2562–2570. doi:10.1111/j.1462-5822.2007.01036.x. 17714514

[11] Boise LH, Collins CM. 2001. *Salmonella*-induced cell death: apoptosis, necrosis or programmed cell death? Trends Microbiol 9:64–67. doi:10.1016/S0966-842X(00)01937-5. 11173244

[12] Britto CD, Wong VK, Dougan G, Pollard AJ. 2018. A systematic review of antimicrobial resistance in *Salmonella enterica* serovar Typhi, the etiological agent of typhoid. PLoS Negl Trop Dis 12:e0006779. doi:10.1371/journal.pntd.0006779. 30307935 PMC6198998

[13] Zaki SA, Karande S. 2011. Multidrug-resistant typhoid fever: a review. J Infect Dev Ctries 5:324–337. doi:10.3855/jidc.1405. 21628808

[14] Klemm EJ, Shakoor S, Page AJ, Qamar FN, Judge K, Saeed DK, Wong VK, Dallman TJ, Nair S, Baker S, Shaheen G, Qureshi S, Yousafzai MT, Saleem MK, Hasan Z, Dougan G, Hasan R. 2018. Emergence of an extensively drug-resistant *Salmonella enterica* serovar Typhi clone harboring a promiscuous plasmid encoding resistance to fluoroquinolones and third-generation cephalosporins. mBio 9:e00105-18. doi:10.1128/mBio.00105-18. 29463654 PMC5821095

[15] Afzal A, Sarwar Y, Ali A, Maqbool A, Salman M, Habeeb MA, Haque A. 2013. Molecular evaluation of drug resistance in clinical isolates of *Salmonella enterica* serovar Typhi from Pakistan. J Infect Dev Ctries 7:929–940. doi:10.3855/jidc.3154. 24334939

[16] Zuckerman JN, Hatz C, Kantele A. 2017. Review of current typhoid fever vaccines, cross-protection against paratyphoid fever, and the European guidelines. Expert Rev Vaccines 16:1029–1043. doi:10.1080/14760584.2017.1374861. 28856924

[17] Syed KA, Saluja T, Cho H, Hsiao A, Shaikh H, Wartel TA, Mogasale V, Lynch J, Kim JH, Excler J-L, Sahastrabuddhe S. 2020. Review on the recent advances on typhoid vaccine development and challenges ahead. Clin Infect Dis 71:S141–S150. doi:10.1093/cid/ciaa504. 32725225 PMC7388714

[18] Johnson MM, Collier MA, Hoang KV, Pino EN, Graham-Gurysh EG, Gallovic MD, Zahid MSH, Chen N, Schlesinger L, Gunn JS, Bachelder EM, Ainslie KM. 2018. In vivo and cellular trafficking of acetalated dextran microparticles for delivery of a host-directed therapy for *Salmonella enterica* serovar Typhi infection. Mol Pharm 15:5336–5348. doi:10.1021/acs.molpharmaceut.8b00802. 30296381 PMC6330710

[19] Hoang KV, Curry H, Collier MA, Borteh H, Bachelder EM, Schlesinger LS, Gunn JS, Ainslie KM. 2016. Needle-free delivery of acetalated dextran-encapsulated AR-12 protects mice from *Francisella tularensis* lethal challenge. Antimicrob Agents Chemother 60:2052–2062. doi:10.1128/AAC.02228-15. 26787696 PMC4808193

[20] Hoang KV, Borteh HM, Rajaram MVS, Peine KJ, Curry H, Collier MA, Homsy ML, Bachelder EM, Gunn JS, Schlesinger LS, Ainslie KM. 2014. Acetalated dextran encapsulated AR-12 as a host-directed therapy to control *Salmonella* infection. Int J Pharm 477:334–343. doi:10.1016/j.ijpharm.2014.10.022. 25447826 PMC4267924

[21] Tiberi S, Du Plessis N, Walzl G, Vjecha MJ, Rao M, Ntoumi F, Mfinanga S, Kapata N, Mwaba P, McHugh TD, Ippolito G, Migliori GB, Maeurer MJ, Zumla A. 2018. Tuberculosis: progress and advances in development of new drugs, treatment regimens, and host-directed therapies. Lancet Infect Dis 18:e183–e198. doi:10.1016/S1473-3099(18)30110-5. 29580819

[22] Moore TW, Sana K, Yan D, Krumm SA, Thepchatri P, Snyder JP, Marengo J, Arrendale RF, Prussia AJ, Natchus MG, Liotta DC, Plemper RK, Sun A. 2013. Synthesis and metabolic studies of host directed inhibitors for anti viral therapy. ACS Med Chem Lett 4:762–767. doi:10.1021/ml400166b. 23956816 PMC3743129

[23] Achtman AH, Pilat S, Law CW, Lynn DJ, Janot L, Mayer ML, Ma S, Kindrachuk J, Finlay BB, Frinkman FSL, Smyth GK, Hancock REW, Schofield L. 2012. Effective adjunctive therapy by an innate defense regulatory peptide in a preclinical model of severe malaria. Sci Transl Med 4:135ra64. doi:10.1126/scitranslmed.3003515. 22623740

[24] Redza-Dutordoir M, Averill-Bates DA. 2016. Activation of apoptosis signalling pathways by reactive oxygen species. Biochim Biophys Acta 1863:2977–2992. doi:10.1016/j.bbamcr.2016.09.012. 27646922

[25] Raveendran R, Wattal C, Sharma A, Oberoi JK, Prasad KJ, Datta S. 2008. High level ciprofloxacin resistance in *Salmonella enterica* isolated from blood. Indian J Med Microbiol 26:50–53. doi:10.1016/S0255-0857(21)01992-7. 18227598

[26] Hooda Y, Sajib MSI, Rahman H, Luby SP, Bondy-Denomy J, Santosham M, Andrews JR, Saha SK, Saha S. 2019. Molecular mechanism of azithromycin resistance among typhoidal *Salmonella* strains in Bangladesh identified through passive pediatric surveillance. PLoS Negl Trop Dis 13:e0007868. doi:10.1371/journal.pntd.0007868. 31730615 PMC6881056

[27] Saha SK. 1999. A highly ceftriaxone-resistant *Salmonella* typhi in Bangladesh. Pediatric Infect Dis J 18:387. doi:10.1097/00006454-199904000-00018. 10223698

[28] Khan MI, Franco-Paredes C, Sahastrabuddhe S, Ochiai RL, Mogasale V, Gessner BD. 2017. Barriers to typhoid fever vaccine access in endemic countries. Res Rep Trop Med 8:37–44. doi:10.2147/RRTM.S97309. 30050343 PMC6034652

[29] Parry CM, Hien TT, Dougan G, White NJ, Farrar JJ. 2002. Typhoid fever. N Engl J Med 347:1770–1782. doi:10.1056/NEJMra020201. 12456854

[30] Finlay BB, Hancock RE. 2004. Can innate immunity be enhanced to treat microbial infections? Nat Rev Microbiol 2:497–504. doi:10.1038/nrmicro908. 15152205

[31] Schwegmann A, Brombacher F. 2008. Host-directed drug targeting of factors hijacked by pathogens. Sci Signal 1:re8. 18648074 10.1126/scisignal.129re8

[32] Lin HH. 2020. Activation of apoptosis by *Salmonella* pathogenicity island-1 effectors through both intrinsic and extrinsic pathways in *Salmonella*-infected macrophages. J Microbiol Immunol Infect 54:616–626. doi:10.1016/j.jmii.2020.02.008. 32127288

[33] Yu C, Du F, Zhang C, Li Y, Liao C, He L, Cheng X, Zhang X. 2020. *Salmonella enterica* serovar Typhimurium sseK3 induces apoptosis and enhances glycolysis in macrophages. BMC Microbiol 20:151. doi:10.1186/s12866-020-01838-z. 32517648 PMC7282050

[34] Brennan MA, Cookson BT. 2000. *Salmonella* induces macrophage death by caspase-1-dependent necrosis. Mol Microbiol 38:31–40. doi:10.1046/j.1365-2958.2000.02103.x. 11029688

[35] Li Z, Zheng Q, Xue X, Shi X, Zhou Y, Da F, Qu D, Hou Z, Luo X. 2016. Pyroptosis of *Salmonella* Typhimurium-infected macrophages was suppressed and elimination of intracellular bacteria from macrophages was promoted by blocking QseC. Sci Rep 6:37447. doi:10.1038/srep37447. 27853287 PMC5112599

[36] Thurston TLM, Matthews SA, Jennings E, Alix E, Shao F, Shenoy AR, Birrell MA, Holden DW. 2016. Growth inhibition of cytosolic *Salmonella* by caspase-1 and caspase-11 precedes host cell death. Nat Commun 7:13292. doi:10.1038/ncomms13292. 27808091 PMC5097160

[37] Cruz DJM, Bonotto RM, Gomes RGB, da Silva CT, Taniguchi JB, No JH, Lombardot B, Schwartz O, Hansen MAE, Freitas-Junior LH. 2013. Identification of novel compounds inhibiting chikungunya virus-induced cell death by high throughput screening of a kinase inhibitor library. PLoS Negl Trop Dis 7:e2471. doi:10.1371/journal.pntd.0002471. 24205414 PMC3814572

[38] Herb M, Schramm M. 2021. Functions of ROS in macrophages and antimicrobial immunity. Antioxidants 10:313. doi:10.3390/antiox10020313. 33669824 PMC7923022

[39] West AP, Brodsky IE, Rahner C, Woo DK, Erdjument-Bromage H, Tempst P, Walsh MC, Choi Y, Shadel GS, Ghosh S. 2011. TLR signalling augments macrophage bactericidal activity through mitochondrial ROS. Nature 472:476–480. doi:10.1038/nature09973. 21525932 PMC3460538

[40] Paiva CN, Bozza MT. 2014. Are reactive oxygen species always detrimental to pathogens? Antioxid Redox Signal 20:1000–1037. doi:10.1089/ars.2013.5447. 23992156 PMC3924804

[41] Tucker KA, Lilly MB, Heck L, Rado TA. 1987. Characterization of a new human diploid myeloid leukemia cell line (PLB-985) with granulocytic and monocytic differentiating capacity. Blood 70:372–378. doi:10.1182/blood.V70.2.372.bloodjournal702372. 3475136

[42] Eguale T, Gebreyes WA, Asrat D, Alemayehu H, Gunn JS, Engidawork E. 2015. Non-typhoidal *Salmonella* serotypes, antimicrobial resistance and co-infection with parasites among patients with diarrhea and other gastrointestinal complaints in Addis Ababa, Ethiopia. BMC Infect Dis 15:497. doi:10.1186/s12879-015-1235-y. 26537951 PMC4634906

[43] Schneider J, Hsieh J, Frantz D, McKnight SL, Ready JM. 2009. Chemical inducers of neurogenesis. US patent 2009/0036451.

[44] Layer RW. 1963. The chemistry of imines. Chem Rev 63:489–507. doi:10.1021/cr60225a003.

[45] Vydzhak RN, Panchishin SY. 2006. Simple synthesis of 1,2-diaryl-1,2-dihydro-chromeno[2,3-c]pyrrole-3,9-diones. Russ J Gen Chem 76:1681–1682. doi:10.1134/S1070363206100331.

[46] Hahn MM, Gunn JS. 2020. *Salmonella* extracellular polymeric substances modulate innate phagocyte activity and enhance tolerance of biofilm-associated bacteria to oxidative stress. Microorganisms 8:253. doi:10.3390/microorganisms8020253. PMC7074811 32070067

[47] Johnson R, Mylona E, Frankel G. 2018. Typhoidal *Salmonella*: distinctive virulence factors and pathogenesis. Cell Microbiol 20:e12939. doi:10.1111/cmi.12939. 30030897

