# Supplementary material for: Identification of a Host-Targeted Compound to Control Typhoid Fever
Source: Microbiol Spectr. 2022 May 17;10(3):e00619-22. doi: 10.1128/spectrum.00619-22 (PMC9241869; doi:10.1128/spectrum.00619-22)
Supplement: SUPPLEMENTAL FILE 1 — Supplemental material. Download spectrum.00619-22-s0001.pdf, PDF file, 0.2 MB [file spectrum.00619-22-s0001.pdf]

Supplemental Figures

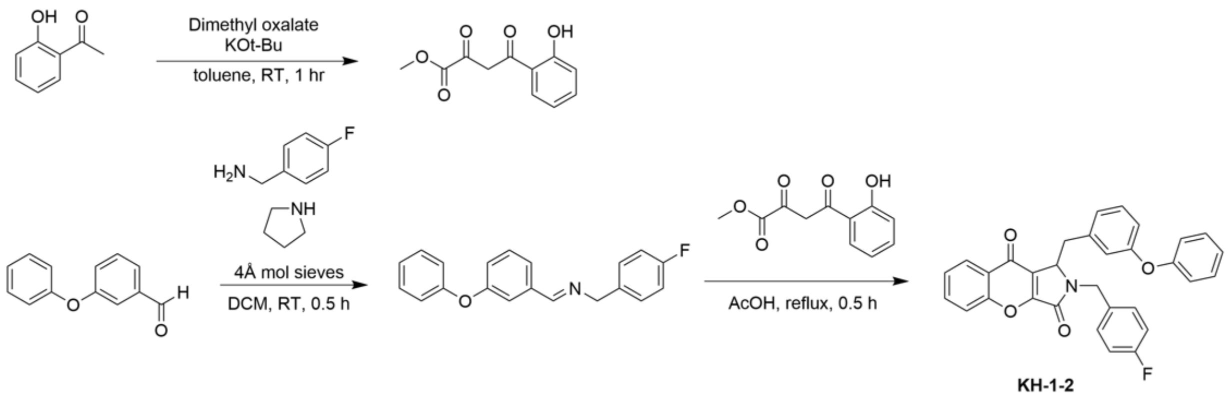

Sup. Fig. 1. KH-1-2 synthesis scheme

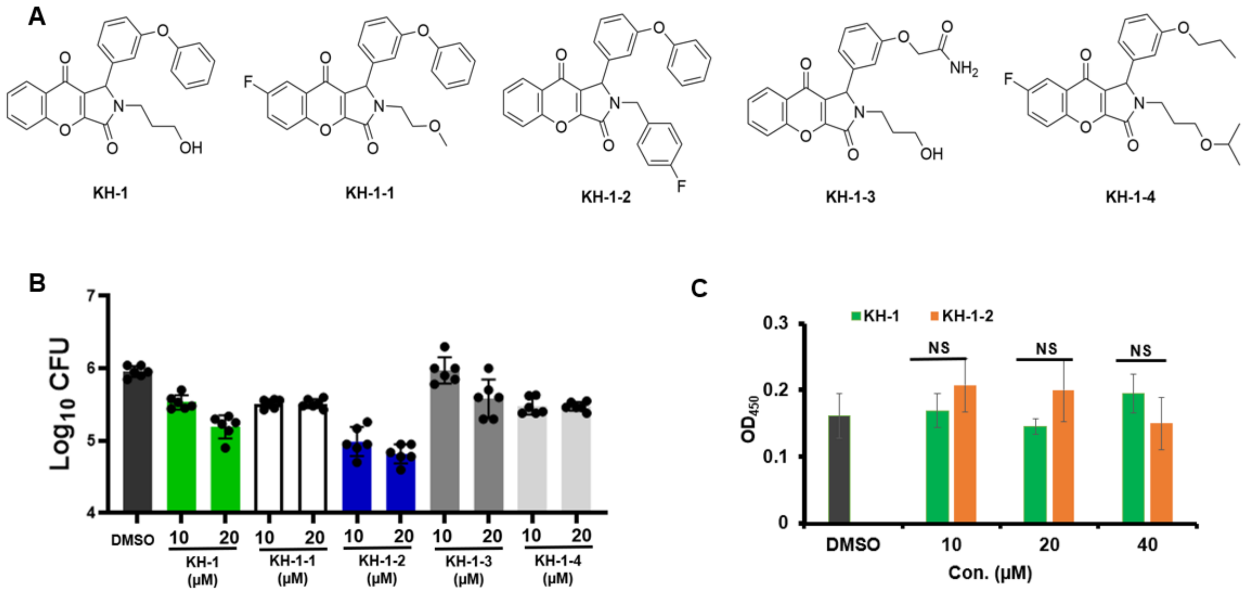

Sup. Fig.2. (A) Chemical structure of four KH-1 analogs. (B) Effects of KH-1 analogs on controlling intracellular *S*. Typhimurium growth inside J774.1 macrophages. *S*. Typhimurium - infected J774.1 macrophages were treated with 10 μM or 20 μM of KH-1 or its analogs, intracellular bacteria were recovered at the indicated time points. (C) KH-1-2 has a similar non-toxic effect on macrophages as KH-1. J774.1 cells were treated with various concentrations of KH-1-2 or KH-1. LDH release was measured at 24 h post-treatment.

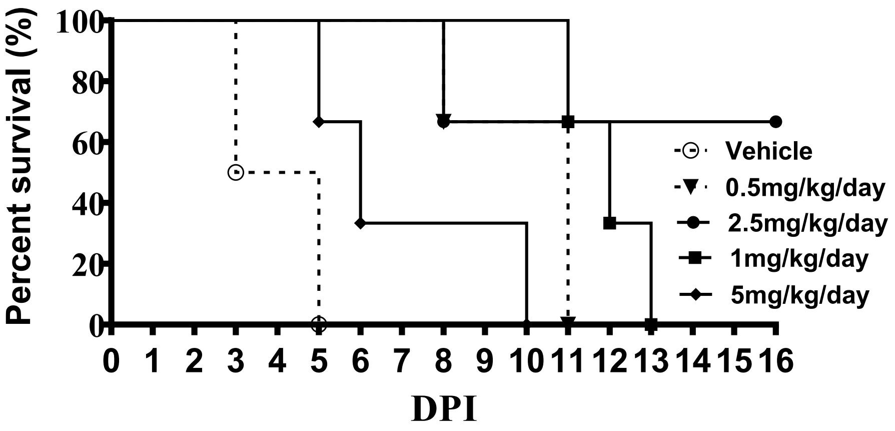

Sup. Fig. 3. KH-1 treatment protects mice from lethal *S.* Typhimurium infection. Mice (4 or 5 mice per group) we orally infected with a lethal dose (10^6 CFU/mouse) of *S*. Typhimurium. One day post-infection, the infected mice were given KH-1 prepared in 200µl PBS at 0.5; 1; 2.5, and 5 mg/kg body weight per day via the intraperitoneal route for 14 consecutive days. The infected mice were monitored for survival for up to 2 weeks post-infection. DPI: days post-infection

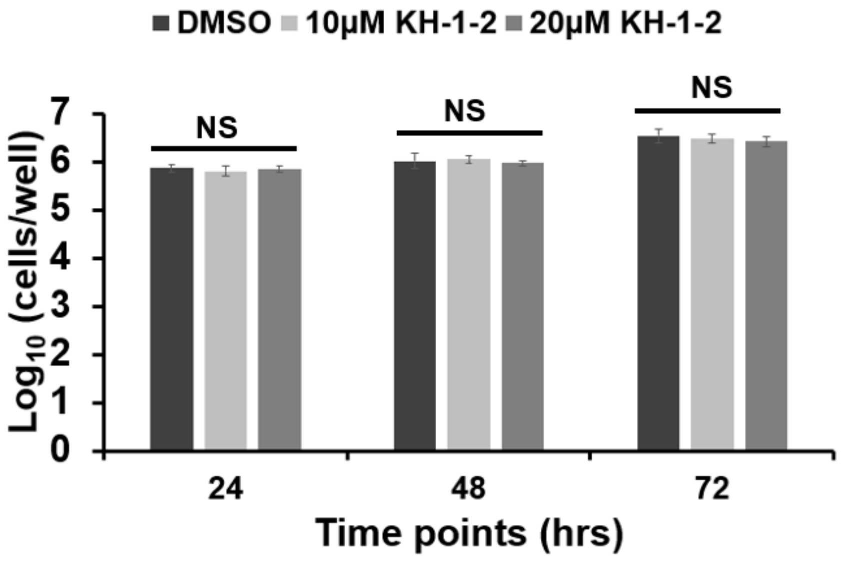

Sup. Fig. 4. KH-1-2 treatment does not affect J774.1 cell proliferation. J774.1 cells were seeded on 6-well plate format at 2 x 10^5 per well and incubated for 3 hrs to allow cells adhere on the plate. The adhered cells were treated with different concentrations of KH-1-2 in triplicate. In the control wells, cells were treated with the equivalent concentration of DMSO. At the indicated time points, the culture medium was removed and wells were washed two times with pre-warmed PBS. The cells then were treated with trypsin and enumerated using an automate cell counter. NS: not significant with respect to DMSO group.

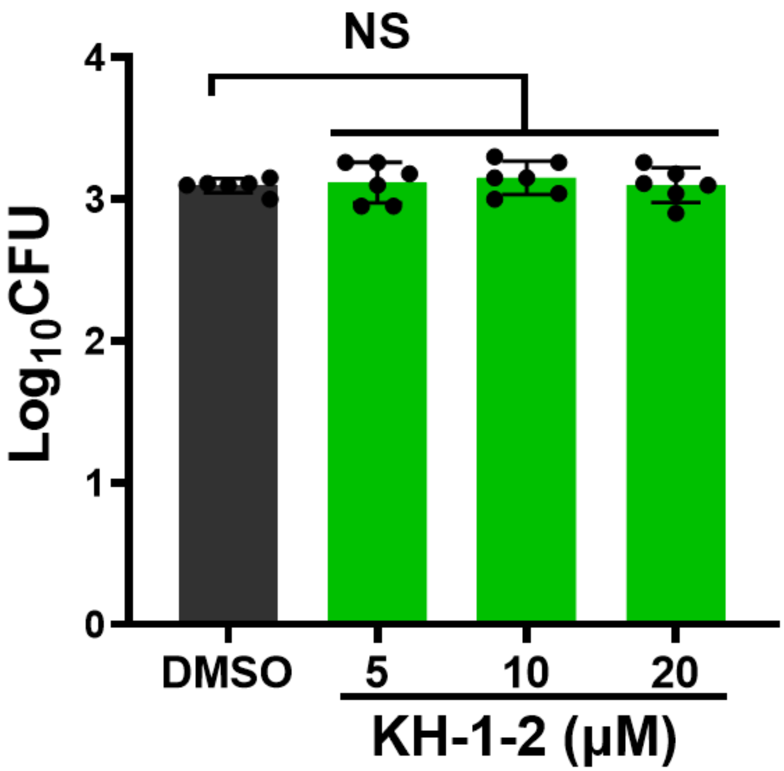

Sup. Fig.5. KH-1 treatment does not affect the invasion and phagocytosis of macrophages by *Salmonella. J774.1* macrophages were treated with various concentrations of KH-1 or DMSO as a control. At 2 hrs post-treatment, the macrophages were washed twice with pre-warmed PBS then infected with *S.* Typhimurium. At 1 h post infection, the extracelluar bacteria were removed by three washes. The bacteria associated with macrophages were recoved by plating. n=3, NS: not significant.
